# Autophagic secretion of HMGB1 from cancer-associated fibroblasts promotes metastatic potential of non-small cell lung cancer cells via NFκB signaling

**DOI:** 10.1038/s41419-021-04150-4

**Published:** 2021-09-22

**Authors:** Yinghui Ren, Limin Cao, Limin Wang, Sijia Zheng, Qicheng Zhang, Xueru Guo, Xueqin Li, Mengmeng Chen, Xiang Wu, Fiona Furlong, Zhaowei Meng, Ke Xu

**Affiliations:** 1 Tianjin Key Laboratory of Lung Cancer Metastasis and Tumor Microenvironment, Tianjin Lung Cancer Institute, Tianjin Medical University General Hospital, Tianjin, 300052 China; 2 Department of Anesthesiology, Tianjin First Center Hospital, Tianjin, 300192 China; 3 The Affiliated Hospital and the Medical College, Hebei University of Engineering, Handan, 056038 Hebei China; 4 Core Facility Center, Tianjin Medical University General Hospital, Tianjin, 300052 China; 5 School of Pharmacy, Queen’s University Belfast, Belfast, UK; 6 Department of Nuclear Medicine, Tianjin Medical University General Hospital, Tianjin, 300052 China

**Keywords:** Cancer microenvironment, Non-small-cell lung cancer, Metastasis, Translational research

## Abstract

Tumor progression requires the communication between tumor cells and tumor microenvironment (TME). Cancer-associated fibroblasts (CAFs) are major components of stromal cells. CAFs contribute to metastasis process through direct or indirect interaction with tumor cells; however, the underlying mechanism is largely unknown. Here, we reported that autophagy was upregulated in lung cancer-associated CAFs compared to normal fibroblasts (NFs), and autophagy was responsible for the promoting effect of CAFs on non-small cell lung cancer (NSCLC) cell migration and invasion. Inhibition of CAFs autophagy attenuated their regulation on epithelial–mesenchymal transition (EMT) and metastasis-related genes of NSCLC cells. High mobility group box 1 (HMGB1) secreted by CAFs mediated CAFs’ effect on lung cancer cell invasion, demonstrated by using recombinant HMGB1, HMGB1 neutralizing antibody, and HMGB1 inhibitor glycyrrhizin (GA). Importantly, the autophagy blockade of CAFs revealed that HMGB1 release was dependent on autophagy. We also found HMGB1 was responsible, at least in part, for autophagy activation of CAFs, suggesting CAFs remain active through an autocrine HMGB1 loop. Further study demonstrated that HMGB1 facilitated lung cancer cell invasion by activating the NFκB pathway. In a mouse xenograft model, the autophagy specific inhibitor chloroquine abolished the stimulating effect of CAFs on tumor growth. These results elucidated an oncogenic function for secretory autophagy in lung cancer-associated CAFs that promotes metastasis potential, and suggested HMGB1 as a novel therapeutic target.

## Introduction

Lung cancer is the most common fatal malignancy worldwide. The most prevalent type of lung cancer is non-small cell lung cancer (NSCLC), it accounts for around 85% of all lung cancer cases [1]. Despite therapeutic advancements, the 5-year survival rate of lung cancer is nearly 15%, in particular, 90% of lung cancer patients die of metastasis, highlighting an urgent need to elucidate the mechanism of lung cancer metastasis.

In recent years, the vital role of tumor microenvironment (TME) in cancer initiation and progression has been recognized. TME promotes both primary tumor and metastatic tumor by contributing to angiogenesis, inflammation, immune regulation, chemoresistance, and invasion [2]. Among the components of TME, such as stromal cells, extracellular matrix (ECM), and blood vascular network, cancer-associated fibroblasts (CAFs) are the majority type of stromal cells. CAFs display higher level of several specific markers compared to normal fibroblasts (NFs), such as α-smooth muscle actin (α-SMA), fibroblast-specific protein 1, fibroblast-activating protein, and tenascin C [3]. Numerous studies revealed that CAFs are involved in tumor progression in various types of cancers, including breast cancer [4], gastrointestinal cancer [5], prostate cancer [6], pancreatic cancer [7], and also lung cancer in our previous study [8].

Autophagy is an evolutionarily conserved catalytic cellular process. By autophagy cells degrade and recycle cellular proteins, lipids, and organelles, providing cells with the source of substrates and energy for maintaining the homeostasis under stresses such as starvation, radiation, and drug treatment [9]. Autophagy is involved in tumor development, in particular, in tumor invasion and metastasis. Interestingly, autophagy plays contrary roles in metastasis. Autophagy may inhibit metastasis by stimulating immune responses and cause tumor cell death. Conversely, it may facilitate tumor cell survival by modulating cells under environment stresses during tumor progression [10]. Recent studies also revealed the effects of autophagy on CAFs. Autophagy plays important roles in CAFs functions including secretion of cytokines, induction of epithelial–mesenchymal transition (EMT), stemness, senescence, and metastasis [11].

High mobility group box 1 (HMGB1) belongs to HMGB family, which consists of HMGB1, HMGB2, and HMGB3. HMGB1 plays multiple roles depending on its location. For example, in the nucleus, HMGB1 regulates gene expression, sustains chromosomal stability, and participates in DNA repair; in extracellular fluid, it interacts with multiple receptors, modulates cell proliferation, inflammation, migration, and autophagy [12]. Accumulated evidences indicate that HMGB1 plays paradoxical roles in both cell survival and cell death through different pathways. In cancer, HMGB1 plays dual roles in both pro- or anti-carcinogenesis by regulating multiple cellular processes such as proliferation, angiogenesis, and invasion [13]. HMGB1 promotes development and metastasis of prostate cancer and colorectal cancer [14, 15]; on contrary, it acts as tumor suppressor in pancreatic cancer and endometrial carcinoma [16, 17]. In the present study, we sought to investigate the role of autophagy in lung cancer patient-derived CAFs. We found that CAFs of lung cancer possess a high basal level of autophagy compared to NFs, and CAFs autophagy activity facilitates the promoting effect of CAFs on lung cancer metastasis via secreting HMGB1.

## Materials and methods

### Reagents and antibodies

3-methyladenine (3-MA), chloroquine (CQ), acridine orange (AO), and rapamycin (RAPA) were obtained from Sigma-Aldrich (St Louis, MO, USA). Human recombinant HMGB1 was purchased from Chimerigen Laboratories (San Diego, CA, USA). HMGB1 neutralizing antibody was purchased from Abnova (Taipei, Taiwan), an isotype control antibody was obtained from R&D Systems (Minneapolis, MN, USA). Glycyrrhizin (GA) was purchased from Topscience (China).

### Cell culture

Human NSCLC cell lines H661 and A549 were obtained from American Type Culture Collection (Manassas, VA, USA). Cells were maintained in DMEM supplemented with 10% fetal bovine serum (FBS) (Gibco, Grand Island, NY, USA) and grew at 37 °C, 5% CO_2_.

### Stromal fibroblast isolation

Fibroblasts were isolated from resected tumor tissues and adjacent non-tumor tissues of patients, who were diagnosed as NSCLC and underwent surgery at Tianjin Medical University General Hospital (TMUGH). Written informed consent was obtained from all patients, and this study has been approved by the Institutional Review Board of TMUGH. CAFs and NFs were characterized as previously described [18]. For the preparation of the conditioned medium (CM), NFs or CAFs isolated from three tissue specimens were pooled and cultured in DMEM/F12 medium supplemented with 10% FBS for 48 h, then the CM was collected.

### Cell proliferation assay

Lung cancer cells were cultured in 96-well cell culture plates. Cells were grown under different treatments for 48 h. Cell proliferation was assessed by a CCK-8 kit, (Dojindo, Kumamoto, Japan), according to the manufacturer’s instructions.

### Detection of acidic vesicular organelles (AVOs)

To detect the formation of AVOs, NFs and CAFs were stained with AO and observed under a Nikon fluorescence microscope (Tokyo, Japan) at ×400 magnification as previously described [19]. Briefly, NFs and CAFs were seeded in 24-well plates and treated with 60 µM CQ for 2 h, then cells were incubated with AO (1 μg/mL) for 15 min.

### Detection of LC3 puncta formation

The LC3 puncta in cells was examined using Ad-GFP-LC3 (Hanbio, China). Briefly, NFs or CAFs were infected with Ad-GFP-LC3. Twenty-four hours after infection, cells were treated with different compounds. The accumulation and distribution of GFP-LC3 puncta were observed under a confocal microscope (Olympus, Tokyo, Japan).

### Cell migration assay

Cell migration ability was assessed by the wound healing experiment as previously described [20]. Briefly, A549 and H661 cells were plated in six-well plates and cultured under different conditions for 24 h. A linear scratch was generated on the cell monolayer using a pipette tip. Photographs of cells were taken by a microscope (Nikon) at ×40 magnification, and the cell migration was assessed by wound closure.

### Cell invasion assay

This assay was performed in 24-well transwell plates as previously described [21]. Briefly, matrigel (BD Biosciences, CA, USA) was diluted six times in DMEM and 40 μL of diluted matrigel was placed on the membrane of the insert. Approximately 1 × 10^5^ cells in serum-free medium were seeded onto matrigel. The inserts were placed into 24-well transwell plates and cultured for 48 h. The invaded cells were stained using 1% crystal violet, and counted under a microscope at ×200 magnification, from ten random fields per well.

### Enzyme-linked immunosorbent assay (ELISA)

The HMGB1 levels in supernatant were detected by ELISA assay. Briefly, medium was collected, and the levels of HMGB1 in medium were detected by ELISA assay as previously described [8]. The concentrations were presented as pg/mL.

### RNA interference and transfection

CAFs were transfected with siATG5 or siControl (GenePharma, Shanghai, China) using Lipofectamine 2000 (Thermo Fisher Scientific, CA, USA). Cell lysates were harvested for western blot to examine the efficacy of siATG5 knockdown. The sequences of siRNA duplex for ATG5 were: sense 5′-GACGUUGGUAACUGACAAATT-3′, antisense 5′-UUUGUCAGUUACCAACGUCTT-3′. The sequences of siRNA duplex for control were: sense 5′-UUCUCCGAACGUGUCACGUTT-3′, antisense 5′-ACGUGACACGUUCGGAGAATT-3′.

### Quantitative PCR (qPCR)

Gene expression was analyzed by qPCR as previously described [22]. Total RNA was extracted from cells using the Trizol reagent (Thermo Fisher Scientific). RNA was reverse-transcribed to cDNA using a TaKaRa Kit (Dalian, China). qPCR was performed using the Power SYBR Green Master Mix (ABI, USA) on an ABI 7500HT Sequence Detector. GAPDH was used as an internal control. Primers were synthesized by BGI (Beijing, China).

### Western blotting

Cell lysates were extracted using RIPA lysis buffer. Cytoplasmic and nuclear extracts were separated using the Nuclear and Cytoplasmic Protein Extraction Kit (Beyotime Biotechnology, China). The cell lysates were separated by SDS-PAGE and transferred onto a nitrocellulose membrane (Millipore, USA). Antibodies against ATG5 (#9980), LC3 (#4108), IΚBα (#9242), p65 (#8242) (1:1000 dilutions, Cell Signaling Technology, USA), E-cadherin (#610182) (1:1000 dilutions, BD Biosciences, USA), TLR4 (#WL00196), p-IΚBα (#WL02495), p-p65 (#WL02169), HMGB1 (#WL03023), PCNA (#WL02208), vimentin (#WL01960), N-cadherin (#WL01047), MMP2 (#WL03224), MMP9 (#WL03096), Twist (#WL00997) (1:1000 dilutions, Wanleibio, China), and β-actin (#ab8224) (1:4000 dilutions, Sigma-Aldrich, USA) were used as primary antibodies. Horseradish peroxidase-conjugated goat anti-rabbit (#ZB-2301) or anti-mouse IgG (#ZB2305) (ZSGB-BIO, Beijing, China) were used as the secondary antibody. The membranes were developed with Chemiluminescent HRP Substrate (Millipore, Bedford, MA, USA) on a G:BOX iChemi XT system (Syngene, Cambridge, UK).

### Xenograft mouse model

Five-week old BALB/c nude mice were purchased from GemPharmatech (Nanjing, China) and maintained under pathogen-free condition. Xenograft experiments were approved by the Tianjin Medical University Institutional Animal Care and Use Committee. Mice were allocated randomly into three groups (*n* = 5/group). To investigate the effect of CAFs on lung cancer cells growth in vivo, 100 µL of A549 cells (3 × 10^6^) alone or mixed with CAFs (6 × 10^6^) treated with/without 60 μM CQ were injected subcutaneously into the flank of mice. The tumor growth was monitored every week. Mice were euthanized after 5 weeks treatment, and tumor tissues were dissected for further analysis. Tumor volume was calculated as: Volume = *d*^2^ × *D*/2, where *D* is the longest diameter and *d* is the shortest diameter.

### Statistical analysis

Data are presented as mean ± SEM (standard error of the mean), and all data were from more than three independent experiments. Statistical analysis between two groups was performed using the Student’s *t* test, and statistical comparisons between groups were analyzed using one-way ANOVA followed by Dunnett’s test for multiple comparisons. The statistical analysis was performed using SPSS21.0. *p* < 0.05 was considered to be a statistical difference.

## Results

### CAFs of lung cancer possess a high basal level of autophagy

**Fig. 1:**
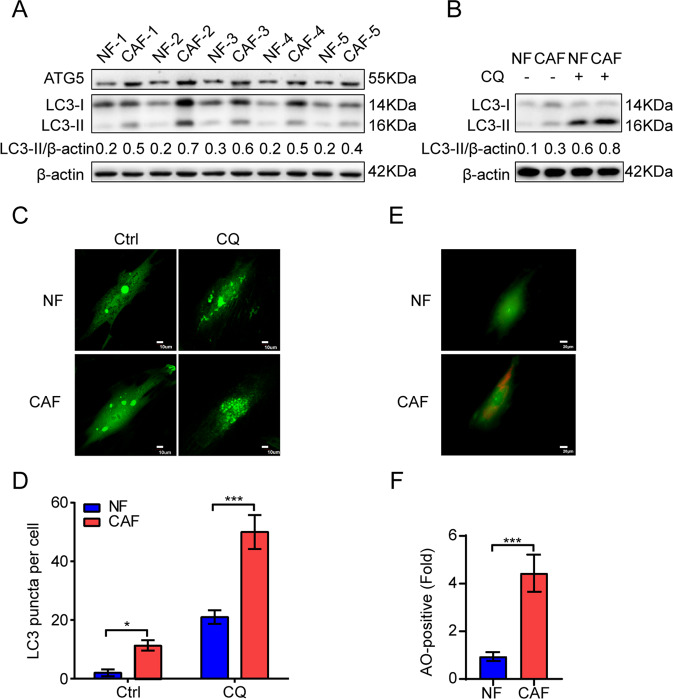
CAFs of lung cancer possess a high basal level of autophagy. **A** The expressions of ATG5 and LC3 in paired CAFs and NFs were detected by western blotting. (*n* = 5). **B** CAFs and NFs were treated with CQ (60 µM) for 2 h, the expression of LC3 was detected by western blotting. **C** CAFs and NFs were infected with Ad-GFP-LC3. After 24 h, cells were treated with CQ (60 µM) for 2 h. LC3 puncta patterns were observed under a confocal microscope. Scale bar, 10 μm. **D** Quantitative analysis of GFP-LC3 puncta. **E** CAFs and NFs were incubated with AO (1 μg/mL) for 15 min. The formation of acidic vesicles organelles (AVOs) was observed under a confocal microscope (×400 magnification). Scale bar, 20 μm. **F** Quantitative analysis of formation of AVOs. Data represent the mean ± SD from three independent experiments. Columns, mean; bars, SD. *p < 0.05, ***p < 0.001. Ctrl control.

Our previous studies showed that CAFs enhance lung cancer cell metastasis, and CAFs are more effective than NFs [8, 21]. The activity of autophagy in CAFs is responsible for their role in tumors, such as tumor stemness and chemoresistance [11]; however, whether autophagy status is responsible for CAFs’ role in lung cancer metastasis is not clear. We first evaluated the autophagy level by detecting the expression of autophagy-related protein ATG5, and the accumulation of autophagy marker LC3-II, in CAFs derived from primary lung tumor tissues and NFs derived from matched adjacent normal lung tissues. We found that both ATG5 expression level and the ratio of LC3-II/actin expression levels were higher in CAFs than in NFs (Fig. 1A). CQ is an autophagy inhibitor, it blocks the fusion of autophagosome with lysosome, which results in the accumulation of LC3-II. Pretreatment with CQ for 2 h also showed the higher ratio of LC3-II/actin expression in CAFs than in NFs (Fig. 1B).

The punctate pattern of LC3 is also widely used to detect autophagy. Both CAFs and NFs were infected with Ad-GFP-LC3 and observed under microscope. Figure 1C, D indicates that CAFs expressed more LC3 puncta than NFs, and CQ treatment showed the similar result. In addition, the formation of AVOs is another hallmark of autophagy. AVOs can be stained with AO and characterized by orange fluorescence. After AO staining, we found that CAFs displayed significant stronger orange fluorescence than NFs (Fig. 1E, F). Taken together, our results indicated that CAFs of lung cancer possess a higher basal level of autophagy compared to NFs.

### The autophagy activity of CAFs facilitates the promoting effect of CAFs on lung cancer cell migration and invasion

**Fig. 2:**
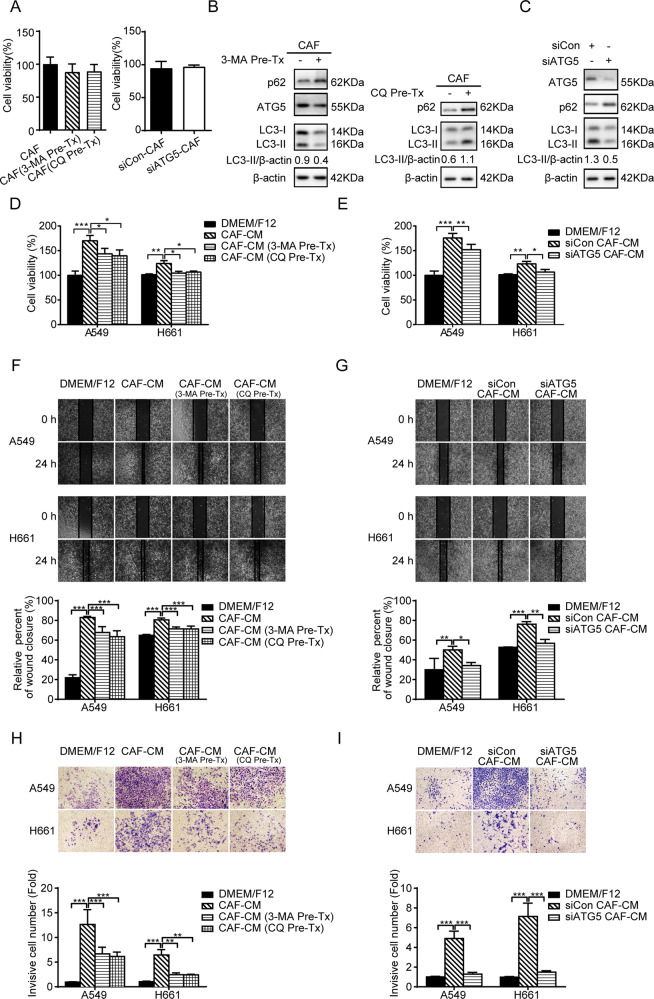
Autophagy inhibition mitigates the promoting effect of CAFs on lung cancer cell migration and invasion. **A** CAFs were treated with 3-MA (5 mM) or CQ (60 µM) for 2 h or transfected with siATG5 for 24 h. Cell viability was examined by CCK-8 assay. **B** CAFs were treated with 3-MA (5 mM) or CQ (60 µM) for 2 h or **C** transfected with siATG5 for 24 h. The expressions of ATG5, p62, and LC3 were detected by western blotting. **D**, **E** CAFs were pretreated with 3-MA (5 mM) or CQ (60 µM) for 2 h or transfected with siATG5 for 24 h. Medium was replaced with fresh medium and CAF-CM was collected after 48 h and added to lung cancer cells. Cell viability was examined. **F**, **G** CAFs were pretreated with 3-MA (5 mM) or CQ (60 µM) for 2 h or transfected with siATG5 for 24 h. CAF-CM was collected after 48 h and added to lung cancer cells. Cell migration was assessed by the wound healing assay. **H**, **I** CAFs were pretreated with 3-MA (5 mM) or CQ (60 µM) for 2 h or transfected with siATG5 for 24 h. CAF-CM was collected and added to lung cancer cells. Cell invasion was assessed by transwell assay. Representative photographs were presented (×400 magnification). Data represent the mean ± SD from three independent experiments. Columns, mean; bars, SD. *p < 0.05, **p < 0.01, ***p < 0.001. Pre-Tx pretreatment.

The role of autophagy in CAFs’ effect on metastasis was investigated by manipulating the autophagy status of CAFs by different approaches. Firstly, autophagy of CAFs was inhibited by using inhibitors 3-MA or CQ. Following 3-MA or CQ treatment at nontoxic dose (Fig. 2A) for 2 h, cells were washed to remove excess extracellular autophagy inhibitors, and fresh medium was added to prepare CAF-CM. Autophagy was also suppressed by ATG5 knockdown and CAF-CM was collected. To verify the inhibitory effect of 3-MA and CQ or ATG5 knockdown, the expressions of ATG5, p62 and ratio of LC3-II/actin expression were examined by western blot (Fig. 2B, C). Notably, we found that autophagy inhibition repressed the promoting effect of CAFs on lung cancer cell proliferation (Fig. 2D, E).

Tumor cell metastasis depends on cell migration ability. The cell migration ability can be determined by wound healing assay. We cultured lung cancer cells with CAF-CM from above settings, and performed wound healing assay after 24 h. As shown in Fig. 2F, G, the stimulating effect of CAFs on lung cancer cell migration was remarkably mitigated when autophagy activity of CAFs was suppressed. The metastasis also depends on cell invasion ability. Lung cancer cell were cultured with CAF-CM, and cell invasion was assessed by transwell assay. When autophagy of CAFs was inhibited by inhibitors or ATG5 knockdown, cell invasion was reduced dramatically (Fig. 2H, I).

**Fig. 3:**
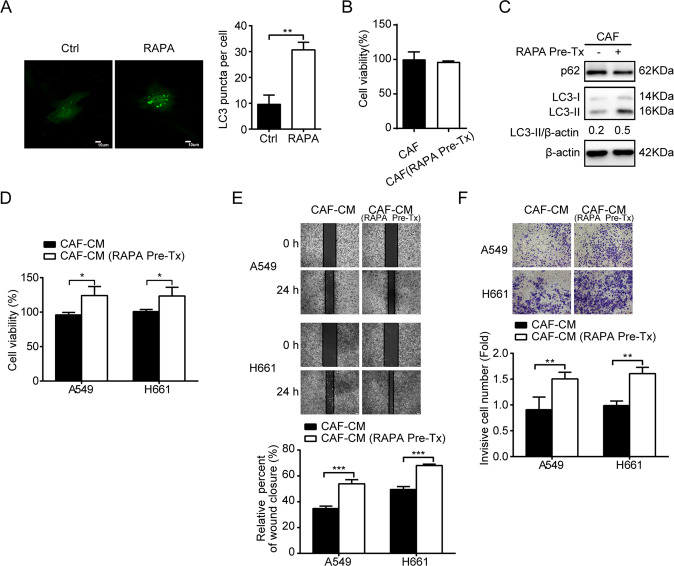
Autophagy activation enhances the promoting effect of CAFs on lung cancer cell migration and invasion. **A** CAFs were treated with RAPA (100 nM) for 2 h. The LC3 puncta patterns were observed under a confocal microscope. Scale bar, 10 μm. **B** CAFs were treated with RAPA (100 nM) for 2 h. CAFs viability was examined. **C** CAFs were treated with RAPA (100 nM) for 2 h. The LC3 and p62 expressions were detected by western blotting. **D** CAFs were treated with RAPA (100 nM) for 2 h. Cell viability was examined. **E** CAFs were pretreated with RAPA (100 nM) for 2 h. Medium was replaced with fresh medium and CAF-CM was collected after 48 h, and added to lung cancer cells. Cell migration was assessed by the wound healing assay. **F** CAFs were treated with RAPA (100 nM) for 2 h. CAF-CM was collected and added to lung cancer cells. Cell invasion was assessed by transwell assay. Representative photographs were presented (×400 magnification). Data represent the mean ± SD from three independent experiments. Columns, mean; bars, SD. *p < 0.05, **p < 0.01, ***p < 0.001. Pre-Tx pretreatment.

To further confirm the role of CAFs autophagy in metastasis promotion, we stimulated CAFs autophagy by using autophagy agonist RAPA. CAFs were treated with 100 nM RAPA for 2 h, then cells were washed and fresh medium was added for CM collection. The elevated autophagy activity of CAFs was determined by punctate pattern of LC3, LC3-II accumulation, and p62 reduction (Fig. 3A–C). In contrast to autophagy inhibition, autophagy activation enhanced the promoting effects of CAFs on both lung cancer cell migration and invasion (Fig. 3D–F). Collectively, our data demonstrated that autophagy activity of CAFs facilitated the promoting effect of CAFs on lung cancer metastasis.

### Inhibition of CAFs autophagy attenuates their regulation on EMT and metastasis-related genes of lung cancer cells

**Fig. 4:**
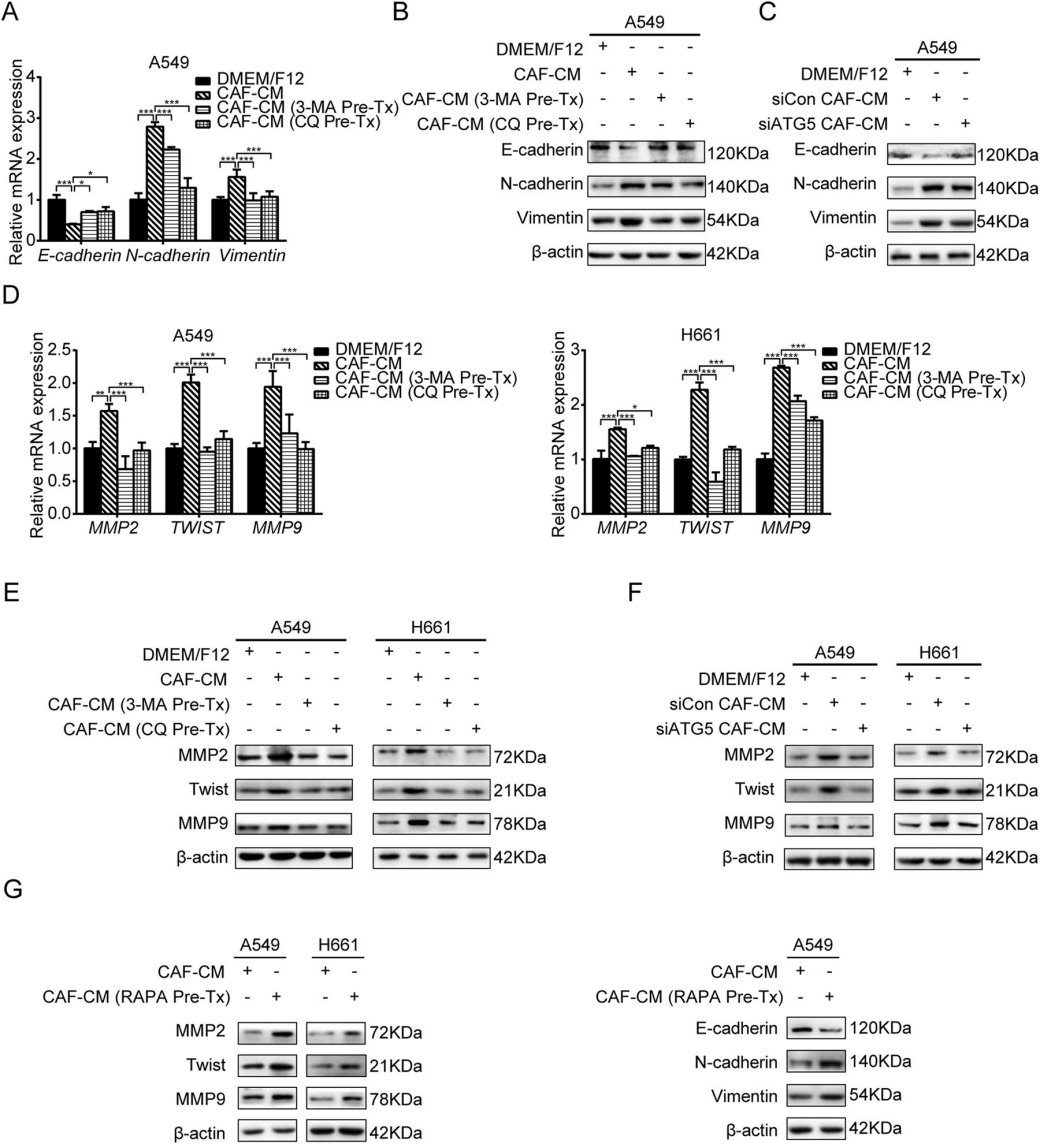
Inhibition of CAFs autophagy attenuates their regulation on EMT and metastasis-related genes of lung cancer cells. **A**–**C** CAFs were treated with 3-MA (5 mM) or CQ (60 µM) for 2 h or transfected with siATG5 for 24 h. Medium was replaced with fresh medium and CAF-CM was collected after 24 h, and added to lung cancer cells. The mRNA and protein expressions were detected by qPCR and western blotting. **D**–**F** CAFs were treated with 3-MA (5 mM) or CQ (60 µM) for 2 h or transfected with siATG5 for 24 h. CAF-CM was collected and added to lung cancer cells. The mRNA and protein expressions were detected by qPCR and western blotting. **G** CAFs were treated with RAPA (100 nM) for 2 h. CAF-CM was collected and added to lung cancer cells. The gene expressions were detected by western blotting. Data represent the mean ± SD from three independent experiments. Columns, mean; bars, SD. *p < 0.05, **p < 0.01, ***p < 0.001. Pre-Tx pretreatment.

EMT is a pivotal step for lung cancer metastasis. During EMT process, bronchial epithelial cells lose cell polarity and cell–cell adhesion, and gain migratory and invasive properties [23]. Our previous study showed that CAFs promote EMT process of lung cancer cells [8]. To clarify whether CAFs autophagy is responsible for lung cancer cell EMT process, we inhibited CAF autophagy by inhibitors or ATG5 knockdown, then collected CAF-CM and cultured lung cancer cells. Compared with untreated CAF-CM, autophagy-inhibited CAF-CM abrogated CAFs’ effect on EMT status of lung cancer cells, evidenced by upregulation of mesenchymal markers N-cadherin and vimentin, downregulation of epithelial marker E-cadherin at both mRNA and protein levels (Fig. 4A–C).

Because metastasis process is regulated by metastasis-related genes, and our previous study revealed that Twist and MMPs are involved in lung cancer cell metastasis stimulated by CAFs [21]. We further treated lung cancer cells with autophagy-inhibited CAF-CM. As shown in Fig. 4D–F, upregulations of Twist, MMP2, and MMP9 by CAFs were significantly reduced by autophagy inhibition of CAFs at both mRNA and protein levels.

Furthermore, we stimulated CAFs autophagy by treating CAFs with RAPA, collected CAF-CM, and examined its effect on EMT status and metastasis-related genes. As shown in Fig. 4G, autophagy activation of CAFs enhanced CAFs’ effect on EMT process and metastasis-related genes expression of lung cancer cells. Altogether, these results demonstrated that autophagy activity of CAFs is essential for CAFs’ effect on EMT and metastasis-related gene modulation of lung cancer cells.

### CAFs autophagy activity regulates HMGB1 secretion

**Fig. 5:**
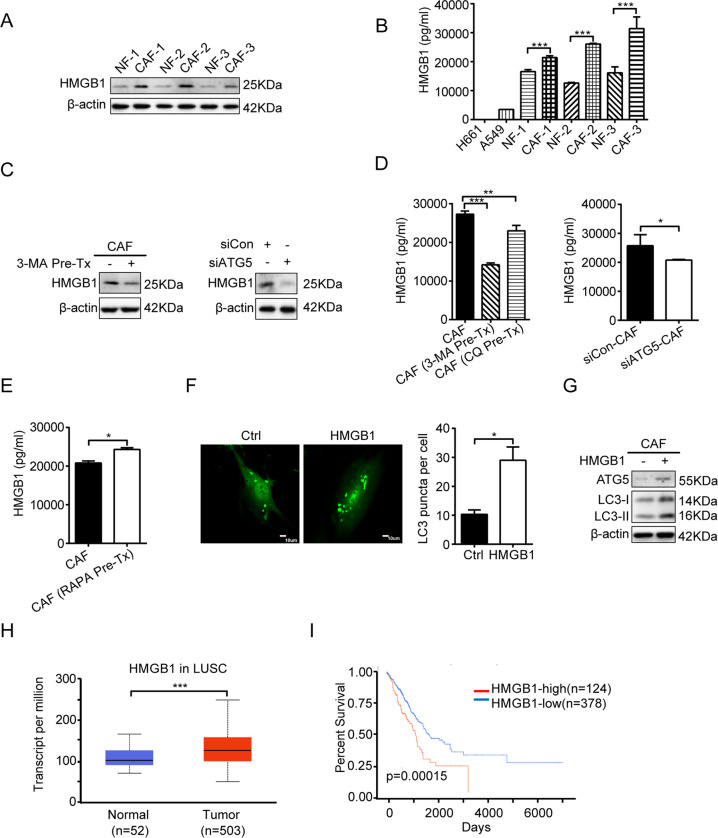
CAFs autophagy activity regulates HMGB1 secretion. **A** The expressions of HMGB1 in paired CAFs and NFs were detected by western blotting (*n* = 3). **B** HMGB1 released by paired CAFs and NFs or lung cancer cells were detected by ELISA (*n* = 3). **C** CAFs were treated with 3-MA (5 mM) for 2 h or transfected with siATG5 for 24 h. The expressions of HMGB1 were detected by western blotting. **D** CAFs were treated with 3-MA (5 mM) or CQ (60 µM) for 2 h or transfected with siATG5 for 24 h. HMGB1 released by CAFs was detected by ELISA. **E** CAFs were treated with RAPA (100 nM) for 2 h. HMGB1 released by CAFs was detected by ELISA. **F** CAFs were treated with recombinant HMGB1 (50 ng/mL) for 48 h. The LC3 puncta patterns were observed under a confocal microscope. Scale bar, 10 μm. **G** CAFs were treated with recombinant HMGB1 (50 ng/mL) for 48 h. The expression of ATG5 and LC3 was detected by western blotting. Data represent the mean ± SD from three independent experiments. **H**, **I** HMGB1 expression level in LUCA, and LUCA patient survival. Columns, mean; bars, SD. *p < 0.05, **p < 0.01, ***p < 0.001. Pre-Tx pretreatment.

The communication between CAFs and tumor cells are mediated via direct or indirect (secretion of proteins) mechanism. Our previous study has compared the gene expression profile between CAFs and NFs derived from lung cancer tissues by RNA sequencing, and we found that HMGB1 is one of the highly expressed genes in CAFs [18]. HMGB1 is a secretory protein, and is reported to be related to cancer initiation, progression, and invasion [13]. To investigate the potential role of HMGB1 in CAFs’ effect, we first detected the endogenous HMGB1 levels in paired NFs and CAFs. We found that CAFs expressed significantly higher level of HMGB1 than NFs (Fig. 5A). Then, we examined the HMGB1 secretion from CAFs by ELISA. Figure 5B reveals HMGB1 level in CAF-CM was higher than in NF-CM. Of note, there were no significant differences in growth speed after 24 or 48 h of incubation between NFs and CAFs (data not shown), thus the HMGB1 were assumed to be released from same amount of CAFs and NFs. Notably, both CAFs and NFs release more HMGB1 than lung cancer H661 and A549 cells.

To clarify whether autophagy affects HMGB1 secretion by CAFs, we suppressed CAFs autophagy by using 3-MA or RNAi knockdown of ATG5. After autophagy inhibition, HMGB1 expressions were dramatically decreased in CAFs (Fig. 5C). Further ELISA assays indicated that autophagy inhibition also significantly reduced HMGB1 secretion in culture medium (Fig. 5D). To further confirm this, we also stimulated CAFs autophagy by using RAPA, as expected, HMGB1 secretion in culture medium was elevated (Fig. 5E).

Numerous studies have indicated the role of HMGB1 in autophagy induction [24]. We therefore assessed the effect of secreted HMGB1 on CAFs autophagy level. Human recombinant HMGB1 were added to CAFs culture medium, and CAFs autophagy level were examined. The LC3 puncta enhancement results indicated that autophagy of CAFs was stimulated, and autophagy induction was further confirmed by LC3-II and ATG5 increase (Fig. 5F, G). These data indicated that the stimulation of autophagy in CAFs is mediated, at least in part, through the autocrine factor HMGB1 released from activated fibroblasts. Collectively, these results suggested a positive feedback regulation between CAFs autophagy activity and HMGB1 secretion.

These findings drove us to further explore the clinical significance of HMGB1 in NSCLC. We focused on lung adenocarcinoma (LUAD), which is a major subtype of NSCLC. By analysis of The Cancer Genome Atlas Database, we found that HMGB1 is downregulated in LUAD patients compared to healthy control, and high level of HMGB1 is significantly associated with poor survival rate (Fig. 5H, I). These data hinted HMGB1 is involved in lung cancer progression.

### HMGB1 mediated the effect of CAFs on lung cancer cell migration and invasion

**Fig. 6:**
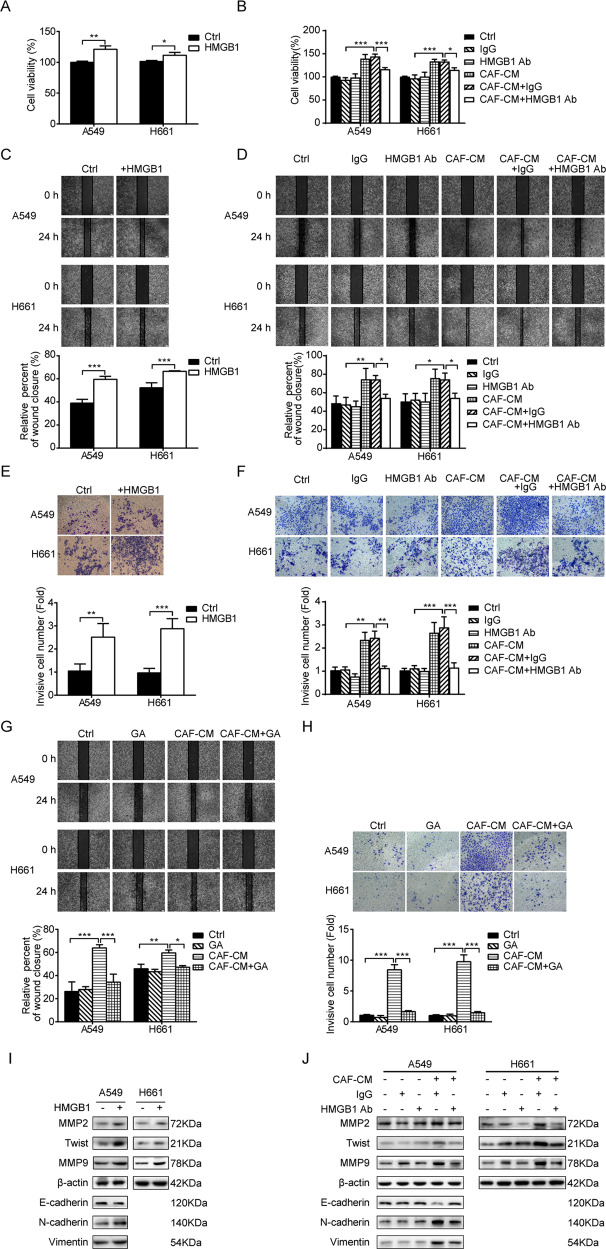
HMGB1 mediates the effect of CAFs on lung cancer cell migration and invasion. **A** Lung cancer cells were treated with recombinant HMGB1 (50 ng/mL) for 48 h. Cell viability was examined. **B** Lung cancer cells were treated with CAF-CM containing HMGB1 neutralizing antibody for 48 h. Cell viability was examined. **C**, **D** Recombinant HMGB1 (50 ng/mL) or HMGB1 neutralizing antibody was added to culture medium. Cell migration was assessed by wound healing assay. **E**, **F** Recombinant HMGB1 (50 ng/mL) or HMGB1 neutralizing antibody was added to culture medium. Cell invasion was assessed by transwell assay. **G** Glycyrrhizin (glycyrrhizic acid, GA) (1 mM) was added to CAF-CM. Cell migration was assessed by the wound healing assay. **H** GA (1 mM) was added to CAF-CM. Cell invasion was assessed by transwell assay. **I** Lung cancer cells were treated with recombinant HMGB1 (50 ng/mL) for 24 h. The gene expressions were detected by western blotting. **J** Lung cancer cells were cultured in CAF-CM containing HMGB1 neutralizing antibody for 24 h. The gene expressions were detected by western blotting. Data represent the mean ± SD from three independent experiments. Columns, mean; bars, SD. *p < 0.05, **p < 0.01, ***p < 0.001. Ctrl control.

In order to investigate the role of CAF-secreted HMGB1 in CAF mediated metastasis, we added human recombinant HMGB1 or HMGB1 neutralizing antibody to culture medium of lung cancer cells, and IgG was used as an antibody control. We first examined their effect on cell viability. HMGB1 showed a moderate stimulating effect on cell proliferation (Fig. 6A). On the other hand, HMGB1 neutralizing antibody had no effect on cell proliferation, however, it blocked the enhancing effect of CAF-CM on lung cancer cell growth (Fig. 6B). The role of CAF-secreted HMGB1 in lung cancer cell migration was assessed by wound healing assay. Recombinant HMGB1 was added to culture medium and Fig. 6C shows that HMGB1 promoted cell migration, whereas, when HMGB1 neutralizing antibody was added to CAF-CM, it abrogated CAF’s effect on cell migration (Fig. 6D). In line with this finding, Fig. 6E, F shows the similar effect of HMGB1 and HMGB1 neutralizing antibody on cell invasion through transwell assay. To further validate our findings, we suppressed HMGB1 activity in CAF-CM by adding GA, a widely used specific inhibitor of HMGB1 [25]. As expected, significant repression of CAFs’ effect on lung cancer cell migration and invasion was observed following inhibition of HMGB1 (Fig. 6G, H).

Furthermore, we investigated the effect of HMGB1 on EMT process and metastasis-related genes. As shown in Fig. 6I, J, HMGB1 enhanced the expression of MMP2, Twist, and MMP9 and promoted EMT programming, whereas HMGB1 neutralizing antibody mitigated these effects. Taken together, these findings demonstrated that CAFs facilitated lung cancer cell metastasis by secreting HMGB1.

### Autophagy-mediated HMGB1 secretion is responsible for CAFs’ modulation on NF-κB pathway in lung cancer cells

**Fig. 7:**
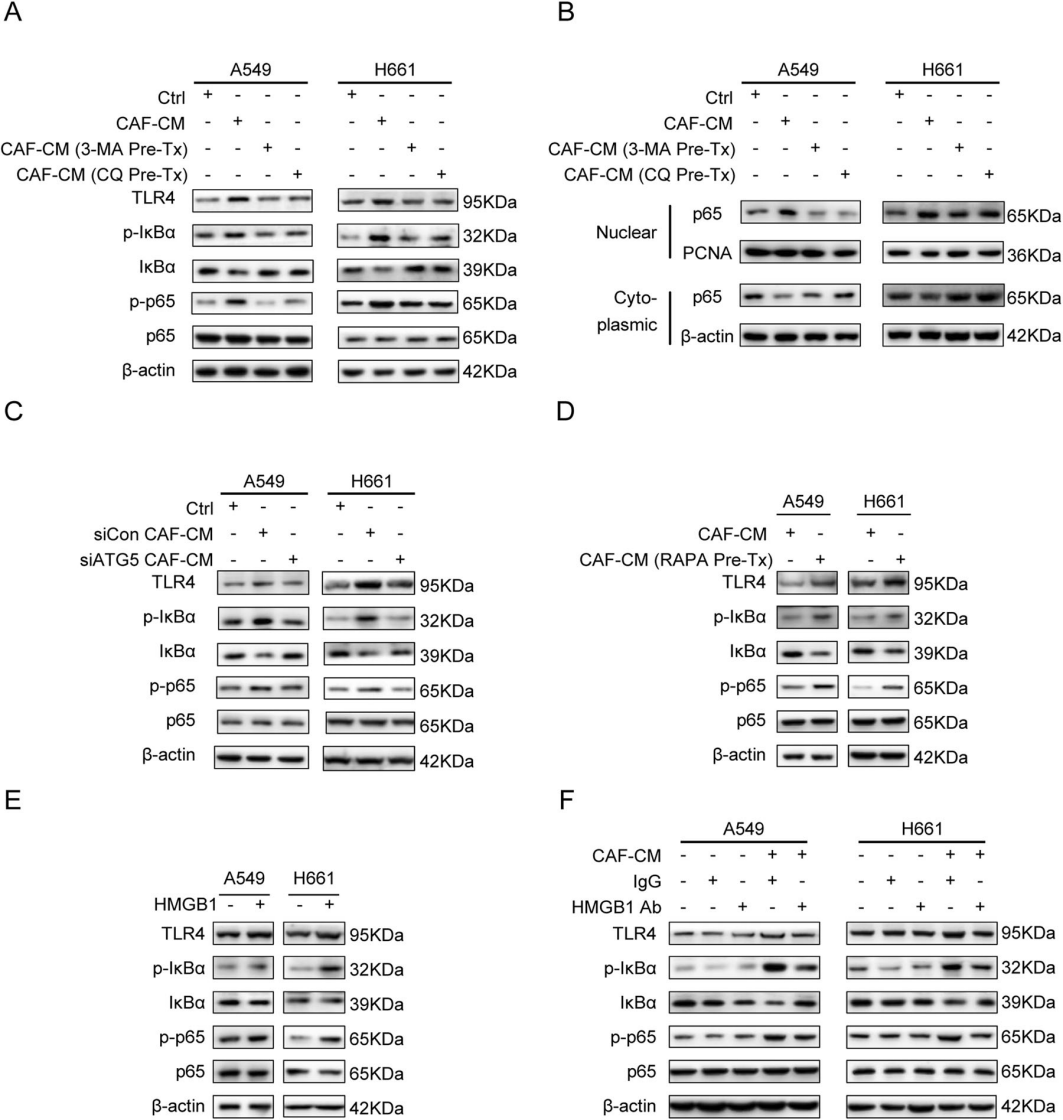
Autophagy activity is responsible for CAFs’ modulation on TLR4/NF-κB pathway in lung cancer cells via HMGB1 secretion. **A** CAFs were treated with 3-MA (5 mM) or CQ (60 µM) for 2 h. Medium was replaced with fresh medium and CAF-CM was collected after 24 h, and added to lung cancer cells. The gene expressions were detected by western blotting. **B** CAFs were treated with 3-MA (5 mM) or CQ (60 µM) for 2 h. CAF-CM was collected and added to lung cancer cells. The expressions of p65 in nuclear compartment and cytoplasmic compartment were detected by western blotting. **C**, **D** CAFs were transfected with siATG5 or treated with RAPA (100 nM). CAF-CM was collected and added to lung cancer cells. The gene expressions were detected by western blotting. **E**, **F** Recombinant HMGB1 (50 ng/mL) or HMGB1 neutralizing antibody was added to culture medium. The gene expressions were detected by western blotting.

TLR4 is one of the cell surface receptors of HMGB1 [26], and TLR4/NF-κB pathway is reported to be responsible for cancer metastasis [27]. To explore the mechanism underlying the role of CAF autophagy in tumor metastasis, we first examined the effect of CAFs on TLR4/NF-κB signaling pathway. After cultured in CAF-CM, the TLR4 expression in lung cancer cells increased, followed by the enhancement of IκBα phosphorylation as well as p65 phosphorylation, meanwhile, total IκBα and p65 remained unchanged (Fig. 7A). NF-κB activation was also determined by p65 nuclear translocation. Figure 7B shows that p65 was increased in nuclear compartment, whereas it decreased in cytoplasmic compartment.

We then suppressed CAFs autophagy by using autophagy inhibitors 3-MA and CQ or ATG5 knockdown. When autophagy was inhibited, the effect of CAFs on activations of TLR4 and NF-κB was mitigated remarkably (Fig. 7A–C). Interestingly, when we stimulated autophagy by using autophagy agonist RARA, we found that RAPA further boosted CAFs’ effects on TLR4 and NF-κB activation (Fig. 7D).

As we have demonstrated that the autophagy activity contributes to CAFs’ effect on the activation of TLR4/NF-κB pathway in lung cancer cells, we further investigated whether HMGB1 is involved in this event. Either recombinant HMGB1 or HMGB1 neutralizing antibody was supplied in cultured medium, and we found that HMGB1 activated TLR4/NF-κB pathway; however, this effect was attenuated by HMGB1 neutralizing antibody (Fig. 7E, F). Collectively, our results show that autophagy-dependent HMGB1 release by CAFs was responsible for stimulating the metastatic potential of lung cancer cells by the activation of the NF-κB signaling pathway, possibly via the interaction of HMGB1 with TLR4.

### Inhibition of autophagy mitigates CAFs’ effect on lung cancer growth in vivo

**Fig. 8:**
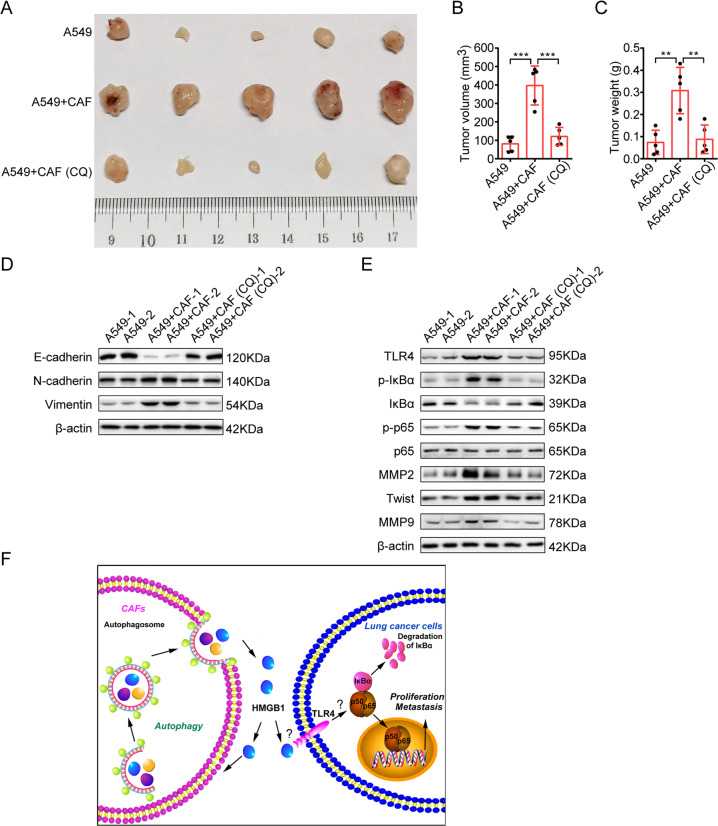
Inhibition of autophagy mitigates CAFs’ effect on lung cancer cell growth in vivo. Lung cancer cells mixed with CAFs treated with/without CQ (60 μM, 24 h) were injected subcutaneously into the flank of mice. Mice were grown for 5 weeks. **A** At the end of the experiment, tumors were excised. **B** Tumor volumes were measured. **C** Tumor weights were measured. **D** EMT marker expressions in tumor tissues were detected by western blotting. **E** The expressions of NFκB pathway genes and metastasis-related genes were detected by western blotting. **F** Schematic mechanism illustrating the role of autophagic secretion of HMGB1 from CAFs on stimulating metastasis of lung cancer cells through TLR4/NFkB axis. Columns, mean; bars, SD. **p < 0.01, ***p < 0.001.

To evaluate the effect of CAFs on lung cancer cell growth in vivo, A549 cells alone or mixed with CAFs were subcutaneously injected into nude mice to establish the xenograft tumor models. We found that CAFs accelerated xenograft tumor growth rate significantly, demonstrated by tumor volume and weight. To further illustrate the role of autophagy in CAFs’ promoting effect on tumor growth, one group of CAFs was also pretreated with autophagy inhibitor CQ. As expected, the inhibition of autophagy by CQ abrogated CAFs’ effect on tumor growth remarkably (Fig. 8A–C).

For the further mechanism study, we performed the western blot analysis on tumor tissues. In line with the in vitro study, we found that CAFs promoted EMT programming, increased metastasis-related genes expression, and activated TLR4/NF-κB signaling pathway. However, when autophagy was inhibited by CQ, CAFs’ effects were attenuated significantly (Fig. 8D, E). These in vivo experiments provided the evidence that the autophagy activity of CAFs is responsible for CAFs’ promoting effect on lung cancer cell growth and metastasis potential.

## Discussion

For many decades metastasis research has been focused on cancer cells; however, recent studies have revealed that TME plays essential roles in metastasis. CAFs are one of the major components of TME, and involved in tumor progress. Our previous studies showed that CAFs enhance lung cancer cell metastasis, and although both NFs and CAFs promote tumor growth, migration, and invasion, CAFs are more effective than NFs. We further demonstrated that CAFs modulate lung cancer cell metastasis through IL-6/STAT3 and KRT8/AKT pathways [8, 21], however, the underlying mechanism is not fully understood.

Cumulative studies have reported that CAFs autophagy not only provide a survival benefit for CAFs in the tumor but also is responsible for the effect of CAFs on cancer cells [28–30]. In the present study, we found that CAFs possess higher level of autophagy than NFs; autophagy inhibition by inhibitors or genetic modulation mitigated CAFs’ effect, but did not block this effect completely, indicating autophagy at least partly contributes to the promoting effect of CAFs on lung cancer cell migration and invasion. Furthermore, our in vivo study showed that inhibition of autophagy by CQ mitigated CAFs’ effect on tumor growth. In our previous study, we examined the CAFs in tumor tissues by detecting α-SMA expression by immunohistochemistry [8]. Our results indicated that the tumor tissues consisted of tumor cells and CAFs, however, the amount of CAFs was much less than tumor cells. Therefore, the reduced tumor size in CQ treatment group was largely due to the reduced amount of tumor cells, it was not because of losing the additional growth of CAFs.

CAFs facilitate tumor development by direct mechanism or indirect mechanism via secreting cytokines and growth factors, such as IL-6 [31], TGF-β^6^, SDF-1 [32], and also exosome [33]. To investigate whether autophagy plays a role in indirect mechanism, we modulated the autophagy activity of CAFs by genetic or chemical inhibition or stimulation, then collected CAF-CM and treated lung cancer cells. We found that the autophagy activity of CAFs has significant influence on CAF-CM’s effect. These findings indicated autophagy regulates CAFs secretion, which is important for tumor metastasis.

Cell secretion is a fundamental cellular process. However, certain leaderless cytosolic proteins cannot enter the classical secretory pathway [34]. They require autophagic machinery for efficient envelopment and exocytosis through an unconventional secretion process [35–37]. Zhao et al. reported that autophagic CAFs-secreted HMGB1 maintains the stemness of breast cancer cells [38]; however, it is still unclear whether HMGB1 released by CAFs via autophagy-mediated secretion facilitates the metastasis of lung cancer cells and tumor growth. Our results showed that autophagy-dependent HMGB1 secretion by CAFs promoted the metastasis of lung cancer cells. Interestingly, we found that CAF-CM had stronger effect than recombinant HMGB1, even though the amount of recombinant HMGB1 was higher than CAF-secreted HMGB1, suggesting there are other factors in CAF-CM that also contribute to CAFs’ effect. Furthermore, we found that CAF-released HMGB1 is responsible, at least in part, for autophagy activation of CAFs, suggesting a positive feedback regulation between CAFs autophagy activity and CAFs secretion, and CAFs remain active through an autocrine HMGB1 loop.

Released HMGB1 binds to cell surface receptors to generate functional responses. There are several HMGB1 receptors are identified, TLR4 is one of these receptors [12]. Recent studies indicate that TLR4/NF-κB pathway is involved in the progress of several types of tumor. Prosaposin promotes the proliferation and tumorigenesis of glioma through TLR4-mediated NF-κB signaling pathway [39]. Cancer cell-derived long pentraxin 3 enhances melanoma migration and invasion through TLR4/NF-κB signaling pathway [27]. Interestingly, quercetin and chrysin inhibit lung cancer invasion and migration through downregulation of TLR4/NF-κB signaling [40]. We also explored the role of TLR4/NF-κB signaling pathway. We first demonstrated the activation of NF-κB signaling. We further investigated the involvement of TLR4, and found that the expression of TLR4 was increased. These findings suggested that NF-κB signaling was involved in CAFs’ effect, and HMGB1 may active NF-κB signaling via TLR4.

In summary, we demonstrated that CAFs-secreted HMGB1 promotes lung cancer cell metastasis, in a CAF autophagy-dependent manner (Fig. 8F), suggesting the potential value of HMGB1 as a novel therapeutic target for lung cancer therapy. As our previous study demonstrated that CAFs promoted lung cancer growth through secretion of IL-6 [8], it is worth to further investigate whether the IL-6 secretion and signaling also depend on autophagy, and whether there is a cross talk between HMGB1 signaling pathway and IL-6 signaling pathway in our future work.

## Data Availability

The datasets used and/or analyzed during the current study are available from the corresponding author on reasonable request.
